# Implementing advance care planning in nursing homes – study protocol of a cluster-randomized clinical trial

**DOI:** 10.1186/s12877-018-0869-1

**Published:** 2018-08-13

**Authors:** Trygve Johannes Lereim Sævareid, Lillian Lillemoen, Lisbeth Thoresen, Reidun Førde, Elisabeth Gjerberg, Reidar Pedersen

**Affiliations:** 1 Centre for Medical Ethics, University of Oslo, Kirkeveien 166 Frederik Holsts hus, 0450 Oslo, Norway; 2 Department of Health Sciences, University of Oslo, Forskningsveien 3A Harald Schjelderups hus, 0373 Oslo, Norway

**Keywords:** Advance care planning, Autonomy, Nursing home, Dementia, Decision-making capacity assessment, Complex intervention, Train the trainer, Mixed-methods

## Abstract

**Background:**

Close to half of all deaths in Norway occur in nursing homes, which signals a need for good communication on end-of-life care. Advance care planning (ACP) is one means to that end, but in Norwegian nursing homes, ACP is not common. This paper describes the protocol of a project evaluating an ACP-intervention in Norwegian nursing homes. The aims of this research project were to promote the possibility for conversations about the end of life with patients and relatives; promote patient autonomy; create a better foundation for important decisions in the case of medical emergencies and at the end of life; and gain experiences in order to find out what characterizes good ACP and good implementation strategies.

**Methods/design:**

This study was a mixed method study including a cluster-randomized clinical trial. Eight nursing home wards or “clusters” were pair-matched, and one ward from each pair was randomly selected for a 12-month intervention. The intervention consisted of implementing an ACP-guideline. Implementation strategies were training and supervision of project teams and staff in using the guideline, written information to patients and next of kin, and information meetings with nursing home staff. The project was evaluated using both quantitative and qualitative data, and both outcome and process evaluation. Quantitative data included patient chart reviews of ACP, diagnoses, patient preferences for decision-making and treatment, values and wishes that are more general, documented life-prolonging treatment and hospitalizations, and concordance between patient wishes and treatment. The primary outcome was documented ACP. Qualitative data included observations of conversations, interviews with patients, next of kin and health care personnel, logs from project coordinators and conversations, and transcripts from meetings with project teams in the intervention group.

**Discussion:**

This project attempted to increase the quality and use of ACP in Norwegian nursing homes (NH). A mixed methods approach, inclusion of patients with dementia, attempts to involve, as many patients as possible, and a sustainable implementation plan adapted to real life in nursing homes were strengths of the project.

**Electronic supplementary material:**

The online version of this article (10.1186/s12877-018-0869-1) contains supplementary material, which is available to authorized users.

## Trial registration

The project is listed on the ISRCTN registry with study ID ISRCTN69571462 – retrospectively registered March 14 2017.

## Background

Close to half of all deaths in Norway occur in nursing homes (NHs) [1]. In such a situation, good clinical communication is required to secure high quality of end-of-life care. However, good communication can be especially challenging in a NH setting where many patients have reduced decision-making capacity (DMC). Advance care planning (ACP) is one way of improving communication. Reports describe improved communication among stakeholders in NHs after use of ACP [2, 3]. A consensus definition of ACP was recently published by a Delphi panel [4]:

“Advance care planning is a process that supports adults at any age or stage of health in understanding and sharing their personal values, life goals, and preferences regarding future medical care. The goal of advance care planning is to help ensure that people receive medical care that is consistent with their values, goals and preferences during serious and chronic illness.”

### Norwegian context

A shortage of staff, competence and time, and a lack of systematic approaches to communication and follow-up of patients and next of kin (NOK) have been identified as important barriers to end-of-life care by Norwegian NH physicians [5]. Yet, in a survey sent to all Norwegian NHs, over half of the managers responded that their NH ‘always’ or ‘in most cases’ did ACP [6]. However, there is reason to believe the practice of ACP varies [6]. Patients participating in ACP in NHs have been observed as quiet, and with a passive, apprehensive attitude [7]. In addition, patients were less likely to take part in ACP than NOK [6]. Patients not participating in ACP is a concern, especially considering many Norwegian NH patients had never talked with their NOK about their preferences for end-of-life care [8]. As a result of not knowing the patient’s preferences, NOK ended up with responsibilities in the decision-making process that they are not comfortable with, nor should have, ethically or legally [9].

### Reduced decision-making capacity

ACP is often not carried out for NH patients with dementia or patients with reduced DMC [10], even though most ACP-programs are considered dementia friendly [11]. Patients with severe dementia may articulate wishes [12, 13]. However, including such patients in ACP as much as possible is both important and challenging. Protecting the patients, and respecting as far as possible their autonomy and capacity [14, 15], requires assessment of DMC for patients known to be at risk for impaired decision-making [16]. Assessment of DMC should be part of every medical encounter, but should be assessed more carefully when 1) there is an abrupt change in mental status, 2) patients refuse recommended treatment, 3) patients consent to particularly risky or invasive treatment too hastily and without careful consideration of the risks and benefits, and 4) patients have a known risk factor for impaired decision-making [16]. The vast majority of NH patients have cognitive impairment and are thus at risk for impaired DMC.

### ACP and patient autonomy

Respect for patient autonomy is now generally favored over paternalistic attitudes and practices that previously dominated the health care services [17, 18]. It is also considered an important goal of ACP [19]. However, freedom and control over medical decisions alone do not constitute patient autonomy [20]. An autonomous person is a person with the capacities of self-governance [21]. Autonomous action is often described as acting intentionally and voluntarily while being informed [21]. The legal and ethical doctrine of informed consent is the main way to respect patient autonomy in health care. A valid informed consent implies information, competence to consent and voluntariness [22, 23]. ACP can make it possible to respect the patient’s autonomy in future treatment, if the patient becomes unable to consent, e.g. due to cognitive impairment. Furthermore, ACP can function as a way to get to know the patient better as an individual. ACP can also contribute to eliciting and respecting other and more general or existential preferences of the patient, e.g. about what is important to the patient to have a good life.

### Project overview

**Fig. 1:**
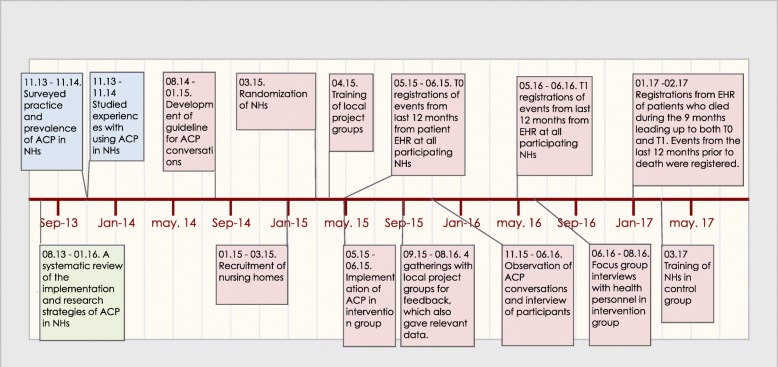
Timeline “End-of-life Communication in Nursing Homes - Patient Preferences and Participation”. Green color indicates part one of the project, blue color indicates part two, and pink color indicates part three

The Centre for Medical Ethics (CME) at the University of Oslo was responsible for carrying out the research project, called «End-of-life Communication in nursing homes. Patient Preferences and Participation». The project consisted of three subprojects (Fig. 1):

#### Subproject 1

Systematic literature review of empirical research dealing with ACP, and decision-making in NHs [10]. The literature review was carried out in cooperation with the COSMOS-study [24].

#### Subproject 2

Mapping present practice in Norway: 2a: The extent of ACP in NHs was surveyed through a questionnaire sent to all Norwegian NHs [6]. 2b: A qualitative study of practice and experiences in Norwegian NHs where some type of ACP was already in place [7, 25].

#### Subproject 3

Based on knowledge and results from subproject 1 and 2, subproject 3 consisted of:

- ACP-guideline to be implemented in NHs
- Training and supervision of project teams that trained and supervised the staff (train the trainers [26, 27]) to be able to use the ACP-guideline
- Written information to patients and NOK
- Information meetings with NH staff
- Evaluation of the influence of the intervention. Exploration of experiences with ACP, the implementation process and intervention among personnel, patients and NOK

This paper presents and discusses the protocol of Subproject 3.

##### Protocol version

Issue date: July 20 2018.

Protocol amendment number: eight.

## Methods/design

### Study design and setting

**Fig. 2:**
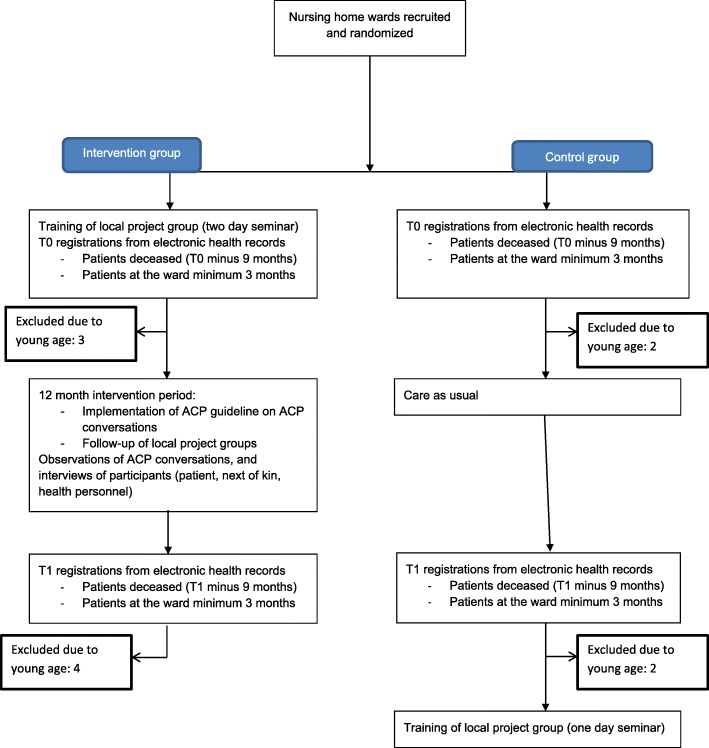
CONSORT flow diagram of the trial

This study made use of a mixed methods approach, including a pair-matched cluster-randomized clinical trial. Clusters were wards at NHs, see Fig. 2 for flow diagram of the trial. 8 wards from 8 NHs were pair-matched based on certain characteristics, using the following data from a national survey/KOSTRA (Municipality-State-Reporting by Statistics Norway)/the NH’s annual report: size of the municipality, size of the NH and ward (number of beds), number of hours with physician present per week, and staff characteristics (educational backgrounds, percentage of professionals, ratio of part time/full time). Patient characteristics did not influence the pair matching. We randomly selected one ward from each pair to an intervention.

### Aim and research questions

In line with the consensus definition of ACP [4], the aims of this research project were to promote the possibility for conversations about the end of life with patients and NOK; promote patient participation/autonomy; create a better foundation for important decisions in the case of medical emergencies and at the end of life; and gain experiences in order to find out what characterizes good ACP, and good implementation strategies in NHs.

Research questions:

- What characterizes ACP in NHs after the implementation of ACP?
- Does an ACP-intervention increase the documentation of patients’ hopes and worries for the future?
- Does an ACP-intervention influence the elicitation of patient preferences about end-of-life issues, such as life-prolonging treatment and hospitalization?
- Does an ACP-intervention increase involvement of patients for decisions on life-prolonging treatment and hospitalization?
- What is the significance of ACP for participating patients, NOK and health care personnel?
- How did we implement the ACP-guideline?
- What are our experiences with implementation of a complex intervention, and what are the barriers and facilitators?

### Complex intervention

ACP is a complex intervention [28]. It requires relatively complex and challenging behaviors, and a certain level of training in and knowledge of the intervention. The intervention targets several groups, and some degree of flexibility in executing the intervention is usually needed. Moreover, there are multiple and variable outcomes involving different stakeholders and levels [28]. The complexity of ACP influences the evaluation of the effect of the interventions [29]. This study attempted to recognize this complexity by involving multiple methods and stakeholders in evaluating the intervention, and by focusing on barriers and promoters in the implementation of the intervention. Process evaluation attempts to evaluate an implementation [30]. In this study, evaluation of the implementation was primarily qualitative [30].

### Recruitment of nursing homes

We included one ward from each participating NH in the study. NHs were chosen from those NHs that in a national survey [6] answered «yes» to the question on interest in participating in this project, and «never/rarely» or «sometimes» to the question of whether they already carried out ACP. Other criteria for inclusion were that the NH and ward management were motivated and had resources to participate in this project.

TJLS contacted NHs by e-mail with an invitation to participate in the study, and then followed-up by telephone. We primarily contacted NH managers. The written invitation contained a definition of ACP, and briefly described the intentions of ACP and possible benefits for the NH to participating in the study. It described aims and design of the study, as well as the randomization process into intervention or control group, the content of the intervention, the need for a local project team at the NH, and how the study would be evaluated. NHs in the study did not receive financial incentives to participate.

### Characteristics of NH wards and patients in Norway

The participating wards were long-term general care wards. In Norway, 13,3% of all Norwegians aged 80 years and older are institutionalized, which for most involves residing in NHs [31]. Among NH patients 82,7% are considered to have extensive needs for assistance in daily living [31] and about 80% have cognitive impairments of different kinds and degrees [32]. NH patients are further characterized by; a mean age of about 84 years, an average of 5–7 diagnoses [32], a high incidence of critical events and critical decisions, and NH patients are admitted to hospital twice as much as the general population [33]. Average stay at NHs until death was about 2 years for patients with a permanent placement [34].

### Research ethics

Participants (patients, NOK, health care personnel) observed in conversations signed an informed consent sheet (Additional file 1). Health care personnel interviewed apart from taking part in observed conversations also signed an informed consent sheet. By signing the sheet, participants accepted researchers observing ACP, and interviewing them after the conversation.

Participants in patient chart reviews were ID numbered. We used the ID number only in the SPSS file. The list containing participants’ names and corresponding ID numbers was stored at the University of Oslo’s ‘Services for sensitive data’ (TSD), which is a platform to store sensitive data in compliance with the Norwegian regulation regarding individuals’ privacy. Consent forms, anonymized transcripts of observations and interviews were also stored in TSD.

Only employees in the project, six researchers and one project coordinator, have access to the data files.

NSD - Norwegian Centre for Research Data (reference number 41114), the Data Protection Official for Research, approved the study.

Furthermore, the regional committee for medical and health research ethics (REC South East) approved (Reference number 2014/2210 REC South East) the patient chart review without consent from patient electronic health records (EHRs) founded in § 18 and § 35 in the Health Research Act [35]. The basis for the approval to access charts without consent was that several of the patients participating in the cluster-randomized clinical trial will lack competency to consent, the potential future benefits of the research e.g. both for patients with and without DMC in NHs, and the low risk. The committee demanded, however, that all patients, or NOK for patients without DMC, received an information sheet that included an opting out opportunity from chart review (Additional file 2). REC South East approved the information sheet.

Permission was granted September 22 2015 by NSD to record sound and observe conversations. Originally, we intended to interview participants of conversations a while after the conversation, and without observing the conversation. By adding observation of conversations to our data gathering, we were hoping to get richer data. Observing conversations would also inform intervention fidelity. In addition, interviewing participants several days or weeks after the conversation would be unwise. Participants may forget relevant information when time passes, perhaps most pertinent for patients with lacking DMC. Interviewing participants within a few hours of the conversation meant content of the conversation would be fresher in mind.

Later, we also received approval to interview bereaved NOK and a hospital physician. In addition, we received approval to do patient chart reviews of patients who died in the period of up to 9 months prior to data collection. REC South East granted both approvals.

### Description of the intervention

#### Clinical intervention and implementation strategies

We targeted the intervention at the health care personnel at the wards in the intervention group. The intervention period started in May 2015 and lasted 12 months. The clinical intervention [36] was implementing systematic ACP.

The implementation strategies included a guideline for how to carry out systematic ACP, local project teams, training, supervision, follow-up, project information and a documentation template.

#### Guideline for how to carry out systematic ACP

We developed a guideline for ACP (Additional file 3). The content and recommendations of this guideline are based on national and international research on ACP, subprojects 1 and 2, and feedback from the project teams at NHs in the intervention group. The guideline includes a definition of ACP, aims and purposes of ACP, emphasizes voluntary participation, addresses DMC, describes the aims of including patients with lacking DMC in conversations, recommends setting and timing of conversation, who should participate and how to prepare for and lead the conversation, what questions to ask, and suggests how the conversations should progress, as well as what to do after the conversation, e.g. documentation and revision.

An invitation template for inviting patients and NOK to conversations is also included in the guideline.

#### Project teams, training, supervision and follow-up

We planned to recruit a local project team from each ward in the intervention group. We recommended that the team consist of 3 people: coordinator, manager from the included ward, and a physician working at the NH (one who is engaged in the subject of ACP). Each ward in the control group was asked to appoint a coordinator.

Choosing a coordinator was meant to be a collaborative effort by researchers at CME and ward management. Criteria for choosing a coordinator: preferably a nurse with legitimacy on the ward; trusted by co-workers; and engaged in end-of-life care and communication. The ward’s coordinator (with the help of the research team) was meant to be responsible for local supervision and training in ACP for the patients’ primary contact persons.

Project teams in the intervention group met for a two-day seminar. At the seminar, we presented the background for the project, how to assess DMC, discussed what ACP is, discussed the guideline for how to carry out ACP systematically, and used role-play to practice (Additional file 4). The teaching followed the «train-the-trainer» model [26, 27]. In this study, this meant that researchers in the project were responsible for teaching, supervision, and follow-up of the ward’s project team. The ward’s project team was then responsible for continued teaching, supervision, and follow-up of relatives, patients, and staff on the wards.

These local project teams also met the researchers throughout the intervention period to discuss the progress of the project. In addition, researchers met staff on the wards to inform about the project, discuss ACP and the guideline with them, and, when time allowed, practice ACP.

#### Project information and documentation template

The implementation plan included providing information material about the project. Distribution of information was in the form of a folder for staff, patients, and NOK, along with a framed poster to hang in the intervention group wards. Furthermore, we developed a website containing information about the project.

Documentation of patient preferences, wishes and values is not only restricted to what is learned from the systematic conversations that define ACP. The ACP process includes spontaneous conversations between health care personnel and patients in everyday situations – labeled by us as ‘golden moments’. Such conversations have by others been called ‘opening the door’ and ‘window of opportunity’ [37]. ‘Golden moments’ may very well be initiated by the patients. To supplement the guideline, and help health care personnel during these spontaneous conversations, we developed a pocket card that includes a few of the questions from the guideline (Additional file 5).

We developed a template for how to document ACP (Additional file 6). The intention of the template was to enable staff to write down the main content of the conversations in a structured way. We developed this to increase the quality of documentation of the conversations. Recommended ACP-content was included in the documentation template.

It is not possible to add the documentation template to the EHRs. Instead, we encouraged project teams and staff on wards to include information noted in the documentation template in the care plan of the patient. The care plan is in the EHR.

### Quantitative data

#### Cluster-randomized clinical trial

The following is a presentation of the pair-matched cluster-randomized clinical trial. We outline outcomes, criteria for inclusion and exclusion, plan for data collection, statistical analysis and power calculation, and sample size.

##### Primary and secondary outcomes

Primary and secondary outcomes are presented in Table 1. We assessed all outcomes using quantitative methods. All information about outcomes is based on patient chart reviews at baseline, and at the end of the 12-month intervention period. We only collected information from those 12 months.

**Table 1:** Primary and secondary outcomes

Primary outcome	
Patients who participated in a conversation on end-of-life treatment	
Secondary outcomes	
Patient’s hopes and worries for the future	
Patient’s wishes for a proxy, information to oneself and NOK, and patient wishes for participation in decision-making processes	
Patient’s competence to consent in relation to conversations on future life-prolonging treatment	
Wishes regarding life-prolonging treatment or hospitalization	
Were these wishes:	
Patient’s own wishes regarding life-prolonging treatment or hospitalization?	
NOK’s knowledge of the patients’ wishes regarding life-prolonging treatment or hospitalization?	
NOK’s own opinion on life-prolonging treatment or hospitalization?	
Patients opting for life-prolonging treatment or hospitalization	
Life-prolonging treatments and hospitalizations	
Life-prolonging treatments and hospitalizations decided against	
Patient’s competence to consent is assessed when life-prolonging treatments or hospitalizations were given or decided against	
Concordance between patient wishes and treatment given	

#### Inclusion and exclusion criteria

We did chart reviews of patients having resided on the participating NH wards 3 months or longer. The minimum time requirement was used so patients had a reasonable chance to participate in ACP, since the guideline recommends ACP within 2 months after NH admission, or earlier depending on the patients’ health.

We collected data from patients who died prior to T0 and T1. We collected the same kind of data for patients who died and those alive.

Patients under the age of 70 and patients who do not know or speak Norwegian were excluded.

### Data collection

We collected quantitative data at T0 (just before the intervention) and T1 (at the end of the intervention period (12 months after T0)). Data from T0 and T1 from patient EHRs in both intervention and control group wards allowed for comparison of the changes in the intervention wards compared to control wards. We developed a data collection form that informed what data was to be collected (Additional files 7 and 8). The data collection form was inspired by a clinical questionnaire to facilitate the process of eliciting patient preferences by Murtagh and Thorns [38] and other previous research on ACP and decision-making on life-prolonging treatment. We collected the same data at T0 and T1.

We collected data on all the participating NH wards for those NH patients that did not opt out.

We aimed to search the EHR for relevant information, but not read everything. Information not found after searching the EHR for about 15 min was not considered readily accessible enough in an acute situation.

We collected data by reading the front page of the patients’ EHR, going through notes written by physicians, nurses and nursing aides, the care plan made by nurses and nursing aides, and all available case summaries, physicians’ referrals and e-links that provide information between NH and hospital after hospitalization.

One NH had started using EHR only a couple of weeks prior to the baseline registrations. As a result information leading up to baseline registrations was primarily handwritten.

### Statistical analysis

We analyzed data using a Generalized Estimating Equations (GEE) approach [39, 40]. The GEE approach was chosen as it is a convenient way of analyzing repeated categorical responses in non-linear models [39]. The cluster sampling strategy in this project challenges the use of traditional regression models. Independent observations are an assumption behind traditional regression models, while cluster sampling in general introduces dependencies. In addition, the project involved repeated measurements on the same individual over time for a number of individuals, which again leads to dependent observations. Overlooking dependencies can lead to a belief that data carry more information than they actually do, by treating each observation as independent [41]. Thus, there is a need for a regression model that can handle dependencies between observations. The GEE approach cannot handle more than one level of dependency. Our design includes clustering of NHs. Thus, we handled dependencies within NHs, and not repeated measurements on the same individual, in the analysis of data. As this is a randomized study, the main analysis was not adjusted for any covariates. However, as a secondary (explorative) approach, we investigated the effect of certain individual level covariates, like cognitive functioning. We used IBM SPSS Statistics 25 in data analysis (IBM Corp.)

### Power calculation and sample size

Based upon similar previous ACP-studies, we assumed the fraction of patients who 3 months (or more) after arrival have participated in ACP would be 10% in group 2 NHs (the control group), and 50% in group 1 NHs (the intervention group) at T1. In order to document this difference of 50–10 = 40 percentage points, with a maximum risk of 5% of committing a type 1 error, and a maximum risk of 20% of committing a type 2 error (i.e. with a statistical strength of .80), our initial sample size calculation showed that we had to include 17 patients from each group. To secure sufficient variability in the NH wards, we concluded that we had to include 4 wards/NHs in the intervention and control arm, each. To adjust for the effect of the clustering of patients and whole ward approach, we did a second sample size calculation, considering the (cluster) design effect. That is, we multiplied the originally estimated sample size with the design effect. The design effect is (1 + (n-1)*ICC), where n is number of observations in each cluster and ICC is intraclass correlation [41]. If we assume *n* = 5 and ICC = 0.02 the design effect is (1 + 4*0.02) = 1.08. 1.08 is the number to be multiplied with 17, giving a sample size of 19 for each cluster. If we assume a bigger ICC of 0.05 there is a design effect of 1.2, giving a sample size of 17*1.2 = 20.4, or 6 patients for each cluster, or 24 patients in total in the 4 intervention wards, and similar numbers in the control wards, or 48 patients in total in the study. Assuming that in both groups 10% of the eligible patients would opt out, we would need to have wards with at least 7 patients on each ward/NH. To make it more likely to have significant results also on the secondary outcomes, we estimated that we had to include at least 15 patients from each ward (in total at least 120 patients in 8 wards/NHs).

### Qualitative data

We gathered all qualitative data from wards in the intervention group. Researchers gathered all data. Qualitative data we studied: experiences gained through ACP by observing ACP, logs from conversations and coordinators, meetings with local project teams, and qualitative interviews. The ward manager or local coordinator identified health care personnel, patients and NOK to be recruited for observations and interviews. Person-identifying details were left out of logs and interview material. Meetings with local project teams, observed conversations and all interviews were audio-recorded and transcribed verbatim.

We assessed fidelity to the intervention informally, based upon our visits, and qualitative interviews/observations. Furthermore, several of the quantitative outcomes can be used as indicators of fidelity.

### Conversation log

A conversation log was sometimes written by the patient’s primary contact person about the execution of ACP and included date, duration, place, participants and content of conversations.

### Meetings with local project teams

Local project teams got together and met researchers. They met on five occasions, one time before the intervention period, three times during and one time after. The purpose of these meetings was primarily implementation support. We also used them to evaluate the implementation strategies, including training and implementation support.

### Coordinator log

The purpose of the coordinator logs was to get descriptions of experiences with the implementation and execution of ACP, and the process over time. The logs included the following themes: How did the coordinator feel about being chosen for the position? Their perceptions about their relationship to other staff members, the teaching by CME, using the guide on the ward, teaching their colleagues, execution of ACP, what happened during conversations, difficult situations, successful situations, how the intervention influences or doesn’t influence cooperation and community on the ward.

### Focus group interviews with staff members

We did focus group interviews with 2–4 health care personnel who participated in ACP on each ward during the intervention period. The topic of the interview was the experiences of being part of these conversations. Examples of interview questions: What is your experience of participating in ACP? What has been difficult, what has been meaningful? What was your role in the conversation/what was your contribution? Are patients who suffer from dementia included in these conversations, and if so, how does that affect the conversation? What is the most important element of the conversation, who is it important for? Does the conversation contribute to patient participation, and if so, how? If for some reason, ACP is not carried out with some patients, how is their ability to participate in end-of-life questions safeguarded? How is the information – and possible patient resolutions - documented and disseminated?

### Observation of conversations

One researcher observed 1–4 conversations on each ward. To learn more of what characterizes ACP, observations focused on the way that the different participants participated in the conversations – who participated, who spoke when, how did they react to topics of conversation, and how did they interact. Observation of conversations inspired the follow-up interviews of participants.

### Qualitative interviews with patients, NOK and health care personnel after ACP conversation

We interviewed participants of ACP within a few hours after the observed conversation. Patients, NOK and health care personnel were interviewed separately. Interviews focused on experiences with taking part in ACP. Examples of interview questions are: Describe the conversation(s) you have participated in. Were you prepared for the topic of the conversation? What did you discuss? How did you like being part of that conversation? Were some things hard to talk about? If you discussed your health, what did you talk about? If you discussed your future, what did you talk about? Did you feel like you were heard and able to participate in important decisions regarding your health? What was your experience of using the guideline during the conversation? Which part of the conversation should be different? How was it for you as NOK to talk about end-of-life issues with the patient present?

Local project teams invited patients to conversations, as indicated in the guideline. If patients agreed, NOK were also invited. When patients were not competent to consent, NOK were invited to conversations to represent the patient.

## Discussion

This protocol follows recommendations for interventional trials [42] according to the SPIRIT checklist.

Randomized controlled trials are considered the best research design to measure effects of an intervention [43]. Still, there are elements to this study that make possible effects harder to assess. Intervention fidelity, inclusion of patients who may lack DMC, possible Hawthorne-effects – wards in the control group may change practice because they know they were observed as part of a research project, and the variation among the participating wards and the staff may influence the effects measured. Measured effects of complex interventions may be misleading as it is hard to determine what caused what. Also, effects of implementation studies are often small or moderate, yet they may still be important [29]. The challenges with measuring effectiveness of complex interventions have led some to propose that trials can only be understood as activity produced through certain contexts [44], making it problematic to generalize the findings. Furthermore, assessing barriers and promoters is a key to evaluating complex interventions and interpreting the findings. The mixed-methods design of the project will add valuable information on the implementation of ACP in NHs, and its possible effects.

In this study, whole wards, rather than individuals, are included, since “real life” implementation often goes through whole wards, rather than individual patients or professionals. Furthermore, we do not exclude patients who have previously participated in ACP, in contrast e.g. to a much cited ACP-study [45]. We also believe that this approach is closer to realities in many modern NH wards since ACP is often implemented already, to some degree. This makes this study differ from many other randomized ACP-trials. If one wants to improve practice and the culture in health care, or to do large-scale implementation of a complex intervention, training existing staff through short training programs is often more sustainable and efficient than using external experts or extensive training of a few selected staff members to do the intervention. ACP is closely interwoven with other tasks and competencies for the professionals in a NH, such as end-of-life communication, getting to know the individual patient, and cooperating with NOK. This is another reason why it is important that most of the existing staff participates in and are trained in ACP. The whole ward approach and cluster design, make it possible to rigorously evaluate the effect of interventions that are likely to “contaminate” or influence many or all patients and staff on a ward, such as training of some staff that work with many patients, or training of most staff. Our intervention includes the whole ward, in contrast to interventions that only influence the individual patients that are included, e.g. as in a drug trial or external facilitators only working with selected patients and only with the intervention. Effect measurements may be affected by including whole wards in this study. More stakeholders will be affected by the intervention (most of the staff and, thus, maybe also most of the patients). However, included patients on wards in the intervention group may not participate in ACP, and included patients may have had ACP prior to inclusion in the study. These factors will contribute to complicate measurements of effect, but will correspond better to real-life health care and what influence to expect from the intervention.

Based on Proctor and colleagues’ descriptions of fidelity as an implementation outcome, it could be argued that this study’s primary outcome (documentation of ACP) measures intervention fidelity [46]. However, documentation of ACP may also be argued to be an indicator of quality of the services (e.g. since patient participation is a legal requirement), and such documentation needs to be available for the professionals practice if they are to be able to follow the patient’s wishes at a later time. Furthermore, it can be argued that such a “process indicator” – i.e. that a systematic conversation has taken place and been documented – is one of the most important outcomes of implementation of ACP, and an important goal in itself, and not only a mean to other ends (e.g. concordance, perceived quality of care, or other service or user outcomes). Finally, we have limited knowledge of how many patients are actually included in ACP when the whole ward approach (which is probably the most common when attempting to improve health care services) is used to implement ACP.

There is a need to improve the quality of and terms and conditions for ACP in Norway [5–7]. This project will contribute to developing better implementation strategies, knowledge of how to perform the implementation of ACP in NHs, and more knowledge of effects, barriers and promoters. The project will give input to how ideals of contemporary health care, in particular respect for patient autonomy [17, 18], can be put into practice in NHs. Respect for patient autonomy is also important for patients with reduced DMC. In contrast to many previous ACP studies [10], this group of patients is included in this study.

The pocket card and documentation template are examples of changes to the project made by the researchers during the intervention period. We have described these and other changes to the project more elaborately in Additional file 9.

### Strength and limitations

One of the strengths of this study is that we randomized participants not on the individual level, but at a cluster level – ward in NH. By doing that, we prevented contamination between the intervention and control group [47]. If each ward had included patients both in the control and intervention group it is more likely patients in the control group (i.e. not getting ACP) could have received some aspects of the intervention, e.g. in other conversations touching upon the same topics. A methodological strength is also that we observed some of the conversations. By observing the conversations, we got first-hand information about how the intervention was implemented on the wards. We could observe verbal and non-verbal expressions during conversations, and got impressions that could give direction to the subsequent interviews [48]. Inclusion of patients with dementia is a strength of the project, as this group of patients is often not included in ACP-studies [10], but recommended in a recent DELPHI study [49]. Inclusion of these patients generates valuable knowledge, since they are important stakeholders in ACP in NHs.

A possible limitation of this study is that the researchers also participated in the implementation, e.g. the training of professionals and in the development of the written information on ACP. Evaluation of effects may be biased if the evaluators are partly responsible for the implementation. However, local staff at the NH carried out the ACP itself, and also the local training and supervision was done by NH-staff (that was trained by the researchers). An advantage of this approach is that the researchers got more detailed knowledge of and experience with the implementation process, and got direct feedback from the professionals that could be used to adjust the implementation. In implementation research, there is often a delicate balance between creating enthusiasm, familiarity and closeness to the intervention and the data, and the search for objective evidence.

Another limitation of the project, is that the local project coordinator on the ward recruited participants to be observed and interviewed. This may lead to recruitment selection bias [47], since the recruiter could avoid recruiting patients and/or NOK that are known to be negative to the NH or the intervention. Furthermore, the professionals most experienced and comfortable with doing ACP may be recruited more often. To not include dose and reach scores as a means of quantitatively evaluating fidelity could be considered a limitation of the project [30]. However, observing conversations and evaluating documentation of ACP could be viewed as an evaluation of compliance to the intervention [46]. In addition, we evaluated the process of implementation of ACP based on meetings with wards in the intervention group, and coordinator logs.

## Conclusion

This project attempts to increase the quality and use of ACP in Norwegian NHs. A mixed methods approach – that includes observations of ACP and a cluster-randomized clinical trial, and inclusion of patients with dementia, attempts to involve as many patients as possible. A sustainable implementation plan adapted to real life at NHs is among the strengths of the project.

Transparency of studies help both clinicians in implementing ACP and researchers in evaluating studies. Detailed descriptions of the protocol, including the implementation strategies, are important to secure transparency in research. Such information secures detailed information on plans, makes it easier to interpret results, and to do similar research and implementation in future. Implementation strategies are, however, often inadequately described in ACP-studies [10]. The basis for this article is thus to describe as fully as possible a mixed method implementation study that includes a cluster-randomized clinical trial.

## Additional files

Additional file 1: Informed consent. (DOCX 734 kb)

Additional file 2: Opting out of chart review sheet. (DOCX 74 kb)

Additional file 3: ACP guideline. (PDF 1839 kb)

Additional file 4: Content of two day seminar. (DOCX 27 kb)

Additional file 5: Pocket card. (PDF 484 kb)

Additional file 6: Documentation template. (DOCX 43 kb)

Additional file 7: Data collection form. (DOCX 24 kb)

Additional file 8: Data collection form – in Norwegian. (DOCX 23 kb)

Additional file 9: Changes to the project. (DOCX 13 kb)

## Abbreviations

ACP: Advance care planning
AD: Advance directive
CME: Centre for Medical Ethics
DMC: Decision-making capacity
EHR: Electronic health record
NH: Nursing home
NOK: Next of kin
