# Supplementary material for: Implementing advance care planning in nursing homes – study protocol of a cluster-randomized clinical trial
Source: BMC Geriatr. 2018 Aug 13;18:180. doi: 10.1186/s12877-018-0869-1 (PMC6090595; doi:10.1186/s12877-018-0869-1)
Supplement: Supplementary file 1 — Informed consent. (DOCX 734 kb) [file 12877_2018_869_MOESM1_ESM.docx]

**Invitation to patients/residents to participate in a research project: Developing, implementing and evaluating collaborative planning of future health care – advance care planning in nursing homes»**

**Background and purpose:**

In the case of serious illness or sudden worsening of a patient’s health, it can be difficult for the patient to express his/her own wishes. Therefore, it is important that the patient speaks to his/her next of kin, doctor and/or nurse before such a situation arises. The purpose of advance care planning is to find out what the patient wants or thinks about, regarding his/her future, and in the case of deteriorating health. Advance care planning will also encompass the patient’s wishes for or thoughts about the end of life. The Centre for Medical Ethics is carrying out a research project where we want to observe advance care planning conversations, as well as interview patients/residents of the nursing home about their experiences with talking about these topics. The research project is funded by the Norwegian Research Council.

**What does participation mean for you?**

Participation in the study means that you give a researcher permission to observe the conversation between you and the doctor/nurse. Participation also means that the researcher will talk to you afterwards, asking about your experiences from the conversation. We want to know what it was about, and how you felt about discussing your future, serious illness and the end of life. These may be difficult topics and you will decide for yourself if there are things you do not wish to talk about. The interview will last 15–20 minutes. Both the observed conversation and the interview will be audio recorded, and we will be taking notes. If you want someone else to participate with you, perhaps a relative, that is fine.

**What will happen to the information about you?**

Personal data and the things you tell us will be stored securely and processed so that you will not be recognizable to others. The things you tell the researcher will be used to understand more about what is important to seriously ill patients and residents of nursing homes. The results of the research will be disseminated through scientific publication. The research project will end March 1^st^, 2018. Anonymized personal data will be stored for future, related studies.

**Voluntary participation:**

Participation in the study is voluntary. You may withdraw at any time without giving a reason. If you wish to withdraw, all information about you will be deleted. If you choose not to participate, or later withdraw, it will have no effect on your relationship to the staff at the nursing home.

If you have any questions, please contact researcher/Dr. Polit. Lillian Lillemoen who heads the research project: mobile 95043228 or email: [lillian.lillemoen@medisin.uio.no](mailto:lillian.lillemoen@medisin.uio.no)

The study has been granted permission by the Norwegian Centre for Research Data.

Permission has also been granted by the institutional (nursing home) management.

**Forespørsel til pasienter/beboere om deltakelse i forskningsprosjektet**

**«Utvikle, implementere og evaluere felles planlegging av framtidig helsehjelp, - forhåndssamtaler på sykehjem***.***»**

**Bakgrunn og formål**: Ved alvorlig sykdom eller plutselig forverring av helsetilstanden kan det være vanskelig for pasienten å uttrykke egne ønsker. Derfor er det viktig at pasienten snakker med sine pårørende, lege og/eller sykepleier før en slik situasjon oppstår. Hensikten med en forberedende samtale er å få vite hva pasienten ønsker eller har tanker om når det gjelder fremtiden, og når helsetilstanden forverres. En forberedende samtale vil også handle om pasientens ønsker eller tanker for livets sluttfase. I forbindelse med et forskningsprosjekt ved Senter for medisinsk etikk ønsker vi å observere gjennomføring av forberedende samtaler, samt intervjue pasienter/beboere på sykehjem om hvordan de erfarer å delta i en samtale om disse temaene. Forskningsprosjektet er finansiert av Forskningsrådet.

**Hva innebærer deltakelse i studien for deg:** Deltakelsen vil innebære at du gir en forsker tillatelse til å være tilstede under samtalen mellom deg og legen/sykepleieren. Deltakelsen vil også innebære og snakke med forskeren etterpå om dine erfaringer fra å delta i samtalen. Vi ønsker å høre hva den handlet om, og hvordan det oppleves for deg å snakke om fremtiden, alvorlig sykdom og livets sluttfase. Dette kan være vanskelige temaer, og du bestemmer selv om det er noe du ikke vil snakke om. Intervjuet vil ta 15 – 30 minutter. Både den observerte samtalen og intervjuet blir tatt opp på bånd, og det vil bli tatt notater underveis. Hvis du ønsker at noen skal delta sammen med deg på intervjuet, for eksempel en av dine pårørende, er det helt i orden.

**Hva skjer med informasjonen om deg?** Personlige opplysninger og det du forteller vil bli lagret forsvarlig og behandlet slik at du ikke kan gjenkjennes av andre. Det du forteller forskeren vil bli brukt til å forstå mer av hva som er verdifullt og viktig for alvorlig syke pasienter og beboere på sykehjem. Resultatet av forskningen vil gjøres kjent gjennom vitenskapelige artikler. Forskningsprosjektet vil bli avsluttet 1.03.2018. Anonymiserte personopplysninger vil lagres videre for oppfølgende studier.

**Frivillig deltakelse:** Det er frivillig å delta i studien, og du kan når som helst trekke deg uten å oppgi noen grunn. Dersom du velger å avbryte, vil alle opplysninger om deg bli slettet. Om du velger å si nei til deltakelse, eller senere trekker deg, vil det ikke få innvirkning på ditt forhold til ansatte på sykehjemmet.

Om du har spørsmål til studien, ta kontakt med forsker/dr. polit Lillian Lillemoen som leder forskningsprosjektet: mobil 95043228 eller mail: [lillian.lillemoen@medisin.uio.no](mailto:lillian.lillemoen@medisin.uio.no)

Studien er tilrådd av Personvernombudet for forskning, Norsk samfunnsvitenskapelig datatjeneste AS.

Det er gitt tillatelse til gjennomføring av forskningsprosjektet fra institusjonens/sykehjemmets ledelse.

**Samtykke til deltakelse i studien**

Jeg har mottatt informasjon om studien, og er villig til å delta.
Jeg samtykker til at forsker kan observere samtalen jeg skal ha med lege/sykepleier
Jeg samtykker til å delta i intervju

----------------------------------------------------------------------------------------------------------------

(Signert av prosjektdeltaker, dato)
