# Supplementary material for: Implementing advance care planning in nursing homes – study protocol of a cluster-randomized clinical trial
Source: BMC Geriatr. 2018 Aug 13;18:180. doi: 10.1186/s12877-018-0869-1 (PMC6090595; doi:10.1186/s12877-018-0869-1)
Supplement: Supplementary file 2 — Opting out of chart review sheet. (DOCX 74 kb) [file 12877_2018_869_MOESM2_ESM.docx]

Research project about Advance Care Planning (ACP).

Information letter to residents and next of kin about their right to deny access to medical records.

The Centre for Medical Ethics at the University of Oslo is carrying out a research project at your nursing home (or the nursing home of your loved one). The project period is April of 2015 to spring of 2016. The project is about advance care planning in nursing homes.

Advance care planning is conversations between residents, next of kin and staff. The conversations are an opportunity for you and your loved ones to talk about the things that are important to you now and in the future. In these conversations, you can convey thoughts and wishes, and whether you are worried or afraid of anything. The conversations will be used to make better decisions for your treatment and care. Within a year, you and your next of kin will be invited to participate in an advance care planning conversation. It is voluntary to participate. Not wanting to participate is not a problem.

The advance care planning project wishes to:

- Promote the resident’s possibility to affect their own treatment and care
- Promote communication between resident, next of kin and staff
- Create a better foundation to base decisions on (regarding end of life or acute medical events)
- Gather experiences about what characterizes good communication about values, wishes, and end-of-life or acute decision-making.

Over this next year, the project’s researchers will interview some residents, next of kin and staff that have participated in advance care planning. A separate information and invitation letter will be distributed regarding interviews, and participation is voluntary.

Researchers will also be going through residents’ medical records in order to register these residents’ wishes for future care and treatment, wishes regarding information and participation in decision making, whether life-prolonging treatment has been given (for instance antibiotics, artificial nutrition), and possible hospitalizations.

If you do not want researchers to look at your medical records, you can deny them access. Please tell …………………., the ward’s project coordinator. Please contact her if you wish to know more about the project.

The study has been granted permission by the Norwegian Centre for Research Data, and by Regional Committees for Medical and Health Research Ethics.

On behalf of the Centre for Medical Ethics, April 16^th^, 2015.

Trygve Johannes Lereim Sævareid, PhD candidate Lillian Lillemoen, Head of project

Tel: 22 85 92 04, Email: [t.j.l.savareid@medisin.uio.no](mailto:t.j.l.savareid@medisin.uio.no)

Forskningsprosjekt om «forhåndssamtaler i sykehjem»

**Informasjonsskriv til beboer og pårørende om rett til reservasjon**

Senter for medisinsk etikk ved Universitetet i Oslo gjennomfører i perioden april 2015 – våren 2016 et forskningsprosjekt på sykehjemmet hvor du/din nærmeste bor. Prosjektet handler om «forhåndssamtaler i sykehjem».

Forhåndssamtaler er samtaler mellom beboer, pårørende og ansatte. Samtalene er en anledning for deg og dine pårørende til å fortelle hva dere er opptatt av nå og fremover, og hva som er viktig for deg. I samtalene kan du formidle tanker om håp eller ønsker, og om du er urolig for eller frykter noe. Samtalene kan legge føringer for fremtidig behandling og pleie.

I løpet av det neste året vil du og dine pårørende bli invitert til å delta i en forhåndssamtale. Det er frivillig å delta, og ønsker du ikke å være med på forhåndssamtale er det helt i orden å takke nei.

Prosjektet om forhåndssamtaler ønsker å:

- fremme beboeres mulighet til å påvirke behandling og pleie av seg selv
- fremme kommunikasjon mellom beboere, pårørende og ansatte
- gi et bedre grunnlag for viktige beslutninger mot livets slutt eller hvis akutte situasjoner oppstår
- samle erfaring med hva som kjennetegner god kommunikasjon om verdier, ønsker og beslutninger i livets sluttfase eller hvis akutte situasjoner oppstår

Forskere i prosjektet vil det neste året intervjue noen beboere, pårørende og ansatte som har deltatt i forhåndssamtale. Egen informasjon og invitasjon vil bli sendt om å delta i intervju, og deltakelse er frivillig.

Forskere vil også ha gjennomgang av beboeres journal for å registrere beboeres ønsker for fremtidig pleie og omsorg, ønsker for informasjon og medvirkning til beslutninger; om livsforlengende behandling er gitt (for eksempel antibiotika, kunstig ernæring) og eventuell sykehusinnleggelse siste året.

Dersom du ikke ønsker at forskere skal se i din journal har du anledning til å reservere deg. Gi beskjed til …………………., avdelingens prosjektkoordinator. Ta også kontakt med henne om du ønsker mer informasjon om prosjektet.

Prosjektet er godkjent av personvernombudet for forskning, og regional komitè for medisinsk og helsefaglig forskningsetikk.

På vegne av Senter for medisinsk etikk, 16.04.15

Trygve Johannes Lereim Sævareid, stipendiat Lillian Lillemoen, prosjektleder

Tlf: 22 85 92 04, E-post: [t.j.l.savareid@medisin.uio.no](mailto:t.j.l.savareid@medisin.uio.no)
