# Supplementary material for: Implementing advance care planning in nursing homes – study protocol of a cluster-randomized clinical trial
Source: BMC Geriatr. 2018 Aug 13;18:180. doi: 10.1186/s12877-018-0869-1 (PMC6090595; doi:10.1186/s12877-018-0869-1)
Supplement: Supplementary file 3 — ACP guideline. (PDF 1839 kb) [file 12877_2018_869_MOESM3_ESM.pdf]

UiO : Faculty of Medicine
University of Oslo

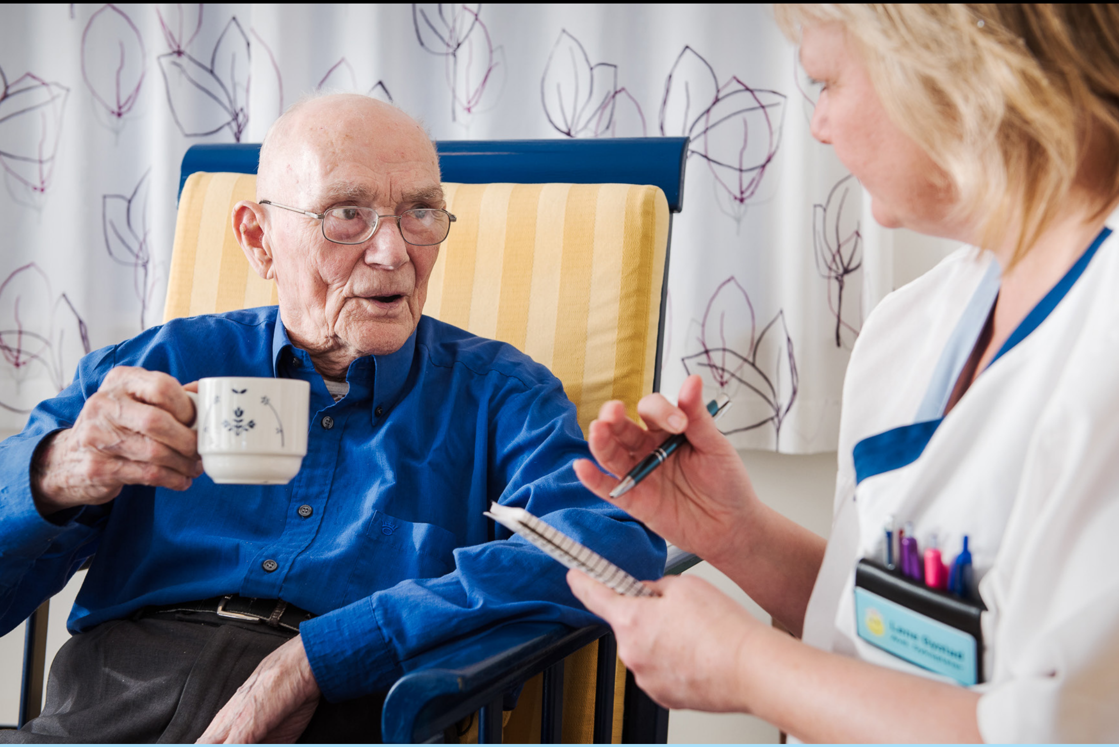

Guide

Advance Care Planning (ACP) – planning for future health care and end-of-life care together

Advance Care Planning (ACP)

Advance Care Planning (ACP) involves the patient and/or next of kin being invited to talk about the patient's near future, health care to come, and the end of life with nursing home staff. They are invited to take part in a conversation some time after the admissions meeting has been held.

ACP can bring valuable information to the staff, but the conversation is, first and foremost, a chance for the patient and relatives to describe what is important to them now, and in the time to come. Using a few concrete questions, the patient is invited to speak about how he/she feels about living in the nursing home, and what is important to him/her.
The patient's responses and wishes should be recorded and should be used as a basis for decisions regarding treatment and care in the future, and at the end of life.

Patients (and relatives) are different, have different life stories, diagnoses, medical histories and experiences, therefore the conversations will all be different.

Consent

Participation is voluntary. Patients who are competent to consent must consent to their next of kin's participation in the ACP. If the patient lacks competence to consent, the conversation should strive to include both patient and next of kin. Our approach is that patients with cognitive impairments can and should be given the chance to relay wishes for their future and their treatment, and be allowed the chance to discuss it with health care staff. At the same time, the conversation's content and extent must be adjusted to fit the patient's condition. The same is true for patients with hearing impairments or aphasia.

The timing of ACP?

Admission meeting

An admission meeting is usually held within 2 weeks of the patient's arrival. The focus in these meetings is usually medical history and mutual orientation about practical matters. Many nursing homes have routines and use templates to guide the conversation in these meetings. Who is next of kin / proxy? Who should be informed about the patient's condition? Other relevant background information?
The admission meeting can also be a preparation for the ACP. This means that health care staff may ask the patient whether he/she wants all the information about their own condition, and the extent to which he/she wants to participate in decisions about medical treatment.

ACP

It is important to carefully consider the timing of the conversation. We recommend that the conversation is carried out within 2 months of arrival, or quicker if the patient's health is very poor. It is an advantage if patient, relatives and staff have had a chance to get to know each other first.

Follow-up

Once or twice a year – topics brought up in ACP-conversations can be brought up, and potentially revised.

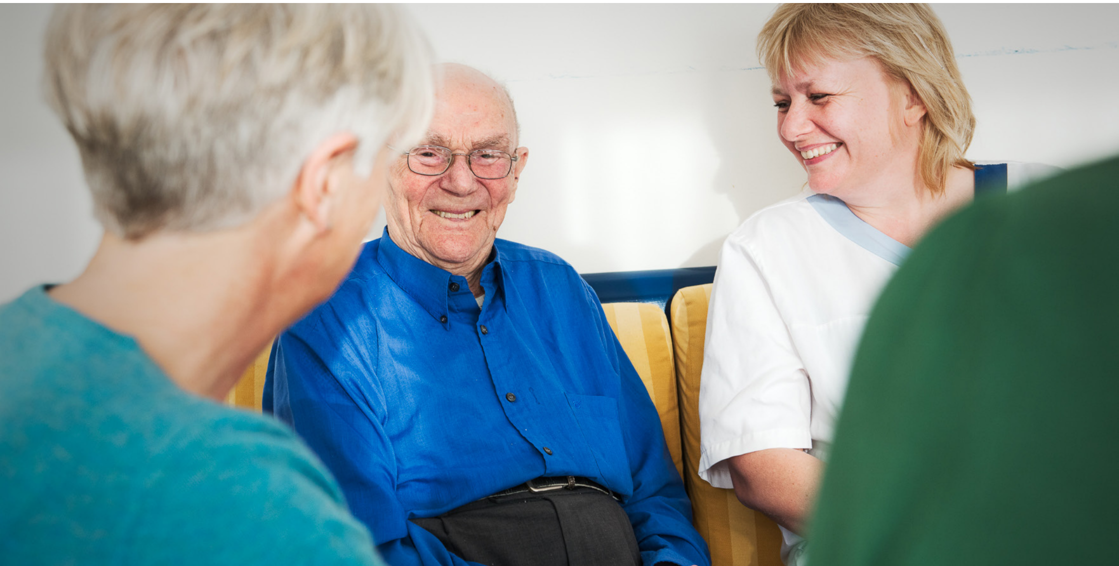

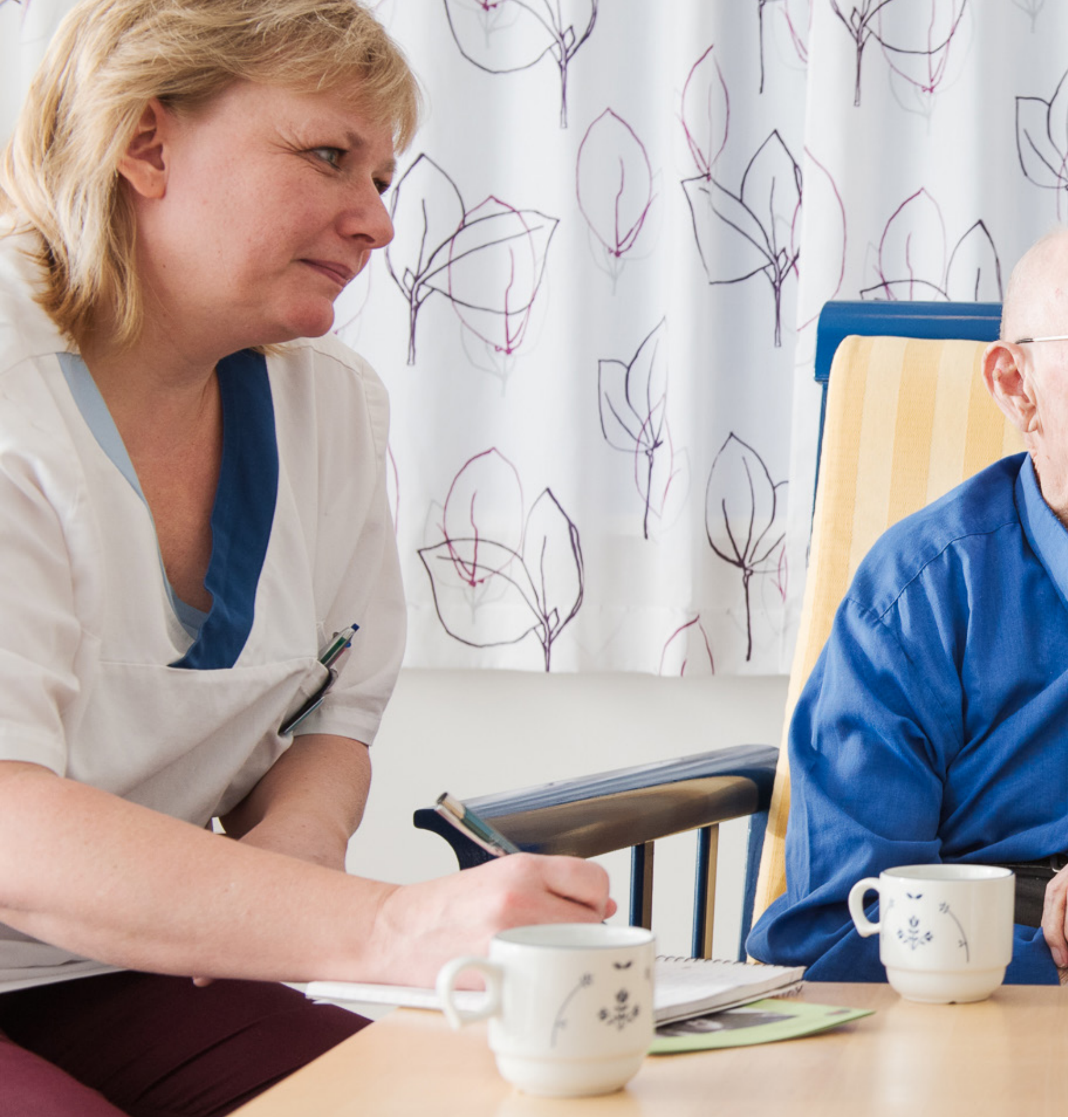

The purpose of ACP

- to further the patient's participation in care and decision-making.
- to foster mutual security and trust between patient and health care staff.
- to give health care staff decision-making support and a better way to move forward.

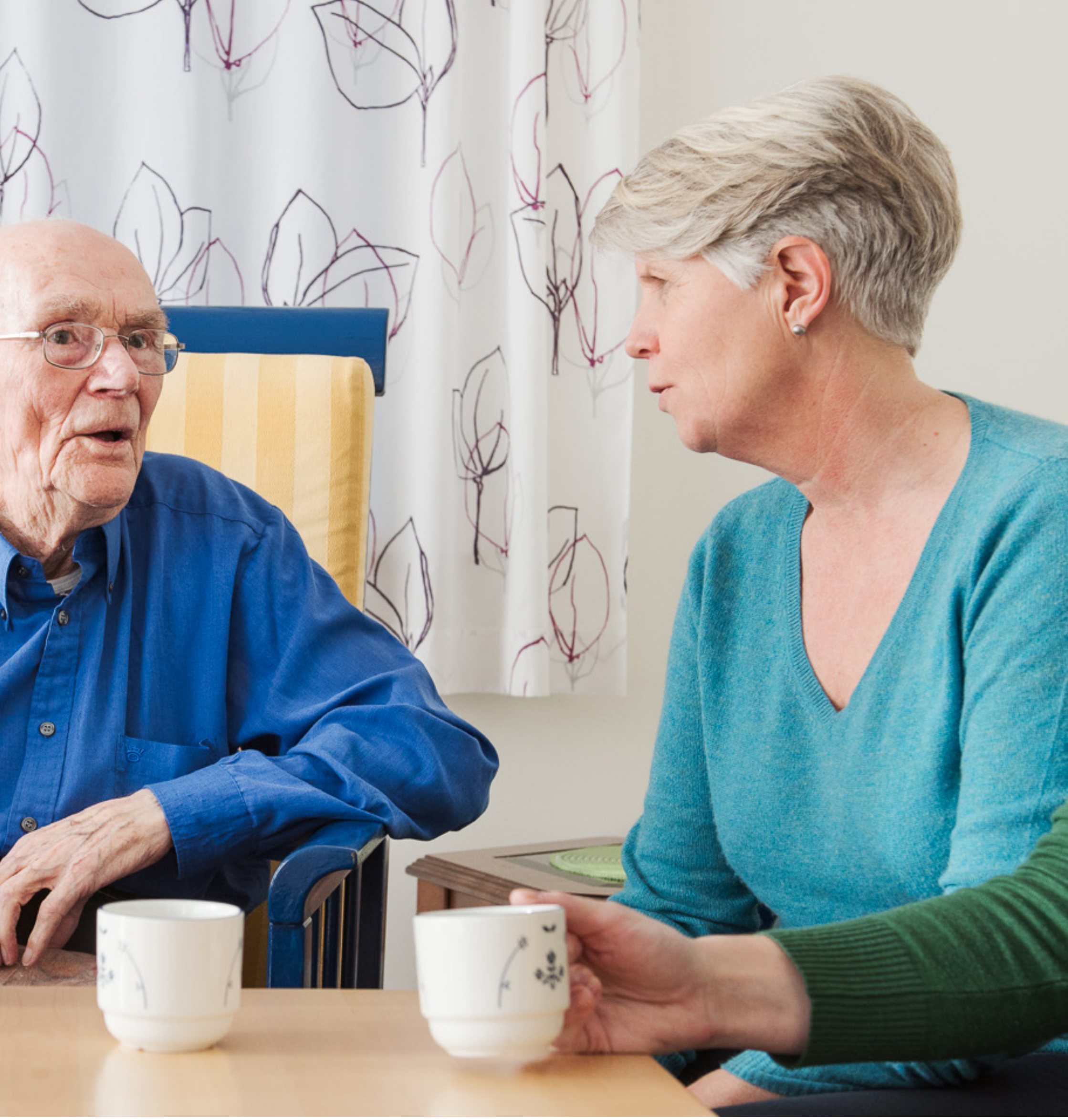

Who should participate?

Patient, next of kin (maybe), the caregiver most familiar with the patient (nurse or nurse assistant) and doctor.

Preparing for ACP

Invitation

Written and verbal invitations to patient and/or relatives. The invitation should make clear the purpose and the content of the conversation, and who should participate.

Time frame

Set aside 45-60 minutes for the conversation, and make this clear in the invitation.

Choosing a room

The conversation should be carried out in the patient's room or other suitable room. Staff common rooms should be avoided, as well as other rooms where people are going in and out, in order to be left alone. Serving coffee or something similar creates a nice atmosphere and makes it feel less formal.

Leading the conversation

The staff should decide before the meeting who will lead it. It may help the patient and/or relatives if you choose someone they are familiar with to lead the conversation, perhaps the caregiver most familiar with the patient.

Patient background

Preparation leads to a more comfortable conversation for those leading it. One way to prepare is to read the patient's chart, background information, or recent daily reports.

Invitation ACP

Here is an example of an invitation. Adjust the text to fit your nursing home.

Dear ……………………………..and relatives/next of kin

You are hereby invited to a conversation with the doctor and a staff member you are familiar with, about how you like living in the nursing home, and any thoughts you may have about the future and the end of your life. We want to get to know you better, and to know more about what is important to you, so that we, as far as possible, can take your wishes and values into consideration. As a patient in the nursing home, you have the right to information and participation in medical decisions. Participation is voluntary; you can turn down this invitation if you wish.

Some of the things we would like to talk about are:

- What is important to you now and in the time to come?
- Do you want to participate in decisions about your medical treatment?
- Have you given any thought to the end of your life, and death?

Many people may feel these are unusual topics to talk about. Therefore, it is a good idea to think them through beforehand, and maybe discuss with your loved ones. Think about whether there are other topics you would like to cover, maybe take a few notes.

If for some reason you are unable to participate, the conversation will be carried out with your next of kin as your proxy. This means that your proxy should relate what is important to you.

The conversation is planned:
Date………………from……………to……………..,
in……………………..

Best wishes,
………………………

Carrying out ACP

The questions need to be adjusted to fit the patient and what information you already have recorded about the patient. It is important to try to create a relaxed atmosphere. For instance, start with small talk based on background information you have, allowing patient, relatives and staff to get comfortable.

It is a good idea to start with some open and more general questions. As the conversation moves along, it is important to be aware and sensitive to the patient's reactions. Relay information in a simple way so the patient understands what he/she is responding to. Point out that it is the doctor who makes the final decisions, but that it is good to know the patient's wishes.

Some of the questions can be hard to answer and require time for thought. Suggested questions to ask the patient:

Suggested beginning questions

- We, the staff, wish to get to know you better, and to know more about what is important to you.
- How is life in the nursing home?
- Who do you spend time with /talk to here in the nursing home?
- Which of your family members and friends stay in touch with you?
- Have you seen the invitation, and have you given any thought to the questions found there?

Suggestions for follow-up questions

- Have you given any thought to the future? Are there things you want to do/experience?
- Do you have any worries?
- Have you ever written down your wishes for future treatment?

Suggested questions regarding future health care

- People handle illness in different ways. Some prefer detailed information about their illness and treatment. Others want more limited information, and others want no information at all. What do you prefer? If you prefer no information, who would you like us to confer with?
- Do you want to participate in decisions regarding treatment and possible hospital admissions? If not, do you want us to confer with your loved ones, and if so, who?
- Some people have thought about what they would like to happen if they suddenly get very sick, if they get pneumonia or their heart stops. Or if they get so sick that hospitalization is necessary. Have you thought about these things? Do you have any requests you think we should know about?
- When you get to the point of your life when you don't have much time left, what is important to you then? For instance, is there someone you want to be with you, or someone you would like to talk to, or anything else that would be important?
- Is there anything else you want to bring up?

We know that some people change their minds about what they would want when they get very sick, therefore we would like to talk to you again about these things later.
Ask the patient and next of kin how it felt to talk through these topics. Set a time for either continuing or finalizing the conversation. Set at time for a follow-up conversation in 6-12 months. Sum up the conversation to make sure everyone has a common understanding of what was decided..

After the conversation

- Document the content of the conversation in the patient's chart.
- If the patient allows it, the content can be conveyed to others who would benefit from the information, for instance family members, family doctor or others.
- The participating staff members should evaluate the conversation: In which ways was it useful? Was it a comfortable conversation from the staff's point of view? How do we think the patient/next of kin felt about it?
- Topics for reflection: Who is this patient and in which ways should the things that came up in the ACP-conversation be taken into our approach?
- Invite the patient/next of kin to give feedback to the nursing home, ideally in writing, about wishes/needs that were not brought up in the conversation.

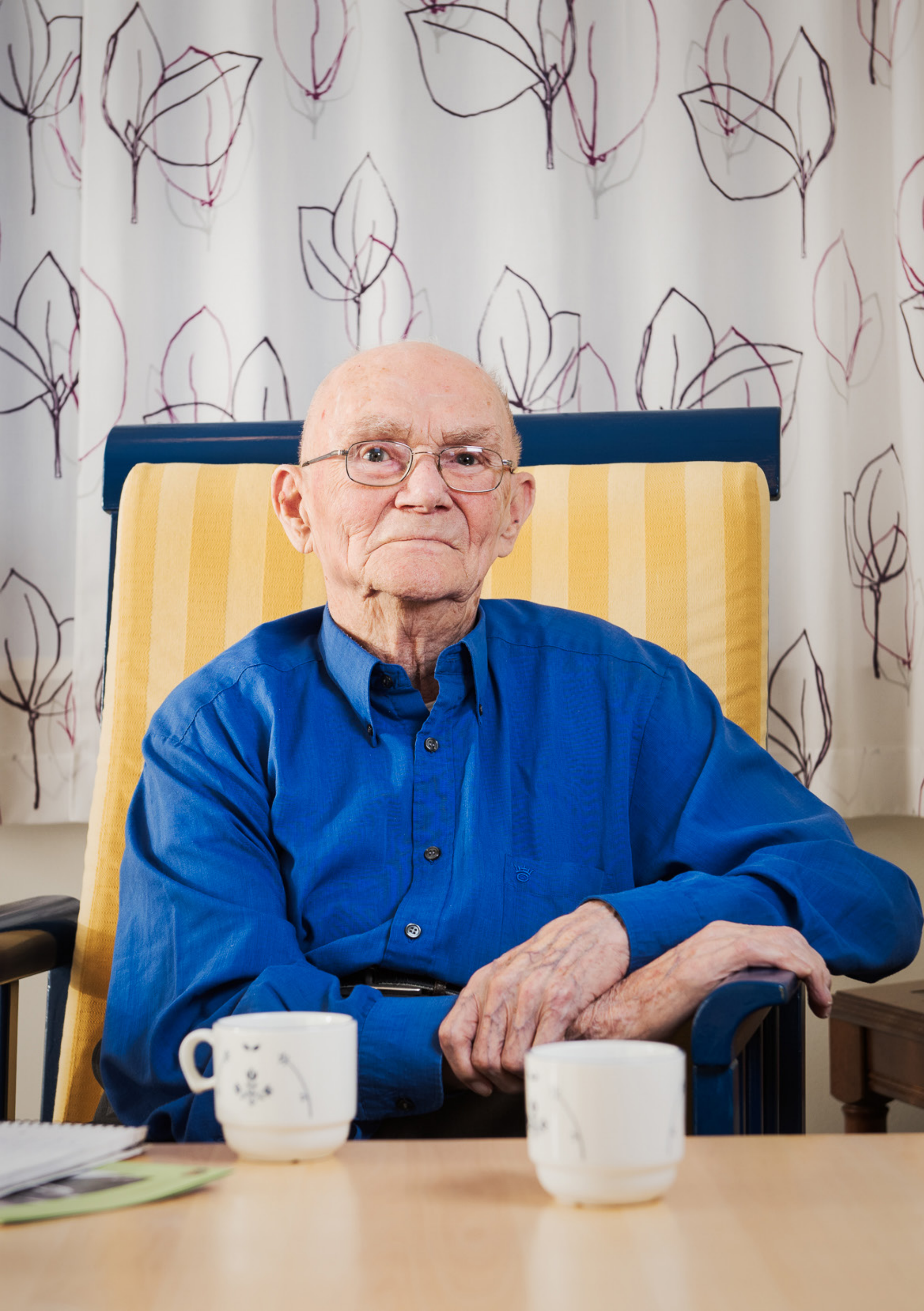

This guide has been tested in the research project: *End-of-life communication in nursing homes. Patient preferences and participation.* The guide will be revised according to the findings and experiences resulting from the project.

The research is carried out by the Centre for Medical Ethics, University of Oslo, in collaboration with the KOSMOS-project at the University of Bergen.

Authors

Lisbeth Thoresen, Lillian Lillemoen, Trygve Johannes Lereim Sævareid, Elisabeth Gjerberg, Reidun Førde og Reidar Pedersen

ISBN 978-82-93544-00-5

UiO : Faculty of Medicine
University of Oslo
