# Supplementary material for: Implementing advance care planning in nursing homes – study protocol of a cluster-randomized clinical trial
Source: BMC Geriatr. 2018 Aug 13;18:180. doi: 10.1186/s12877-018-0869-1 (PMC6090595; doi:10.1186/s12877-018-0869-1)
Supplement: Supplementary file 4 — Content of two day seminar. (DOCX 27 kb) [file 12877_2018_869_MOESM4_ESM.docx]

# Content of two-day seminar

- Presentation of Center for Medical Ethics
- Background for the research project – presentation of the survey, literature review, and qualitative study
- What is ACP?
- The purpose of the conversations?
- The guideline:
  - Who should participate in conversations?
  - When? Follow-up of patients.
  - How? Form and content. Suggested formulations.
  - Documentation and dissemination
- The relationship between ACP and advance directives
- Proxy consent
- Assessment of competency to consent
