# Supplementary material for: Implementing advance care planning in nursing homes – study protocol of a cluster-randomized clinical trial
Source: BMC Geriatr. 2018 Aug 13;18:180. doi: 10.1186/s12877-018-0869-1 (PMC6090595; doi:10.1186/s12877-018-0869-1)
Supplement: Supplementary file 5 — Pocket card. (PDF 484 kb) [file 12877_2018_869_MOESM5_ESM.pdf]

UiO : Det medisinske fakultet

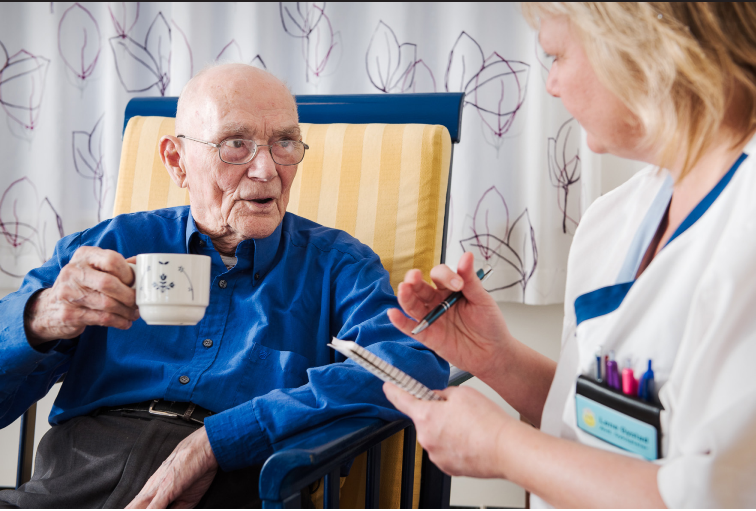

Spørsmål om fremtidig helsehjelp

Spørsmålene må tilpasses pasienten og hvilken informasjon man allerede har dokumentert om pasienten.
Avslutt med å oppsummere samtalen for å sjekke ut en felles forståelse av det som er kommet frem.

Forslag til spørsmål

- Mennesker håndterer egen sykdom ulikt. Noen foretrekker detaljert informasjon om sykdom og behandling, noen ønsker begrenset informasjon mens andre foretrekker å vite lite. Hva foretrekker du? Hvis du ikke vil ha informasjon, hvem skal vi snakke med?
- Vil du være med å bestemme hva du skal ha av behandling og om du eventuelt skal legges inn på sykehus? Hvis ikke; ønsker du at vi skal snakke med en av dine nærmeste, og i tilfelle hvem?
- Noen har gjort seg opp tanker om hva de ønsker hvis de blir akutt eller alvorlig syke; hvis de får lungebetennelse eller hjertet slutter å slå, eller hvis de skulle bli så syk at sykehusinnleggelse er aktuelt. Har du gjort deg noen tanker om dette? Har du noen ønsker her og nå som vi skal vite om?
- Når du kommer dit i livet at du ikke har lenge igjen å leve, hva er viktig for deg da? Er det f.eks noen du ønsker å ha hos deg eller noen du ønsker å snakke med, eller er det noe annet du er opptatt av?
- Er det noe annet du ønsker å ta opp og snakke om?
