# Supplementary material for: Implementing advance care planning in nursing homes – study protocol of a cluster-randomized clinical trial
Source: BMC Geriatr. 2018 Aug 13;18:180. doi: 10.1186/s12877-018-0869-1 (PMC6090595; doi:10.1186/s12877-018-0869-1)
Supplement: Supplementary file 6 — Documentation template. (DOCX 43 kb) [file 12877_2018_869_MOESM6_ESM.docx]

**Documenting Advance Care Planning**

Present in the conversation:

**The patient’s capacity to consent** regarding these question (ie. Yes, no, not assessed):

Comments**:**

***Anything the patient wishes to experience?***

***Does the patient have any worries for the future?***

***The patient’s wishes regarding information about themselves*** (ie. Knowing as much as possible, limited information, does not want to know, unclear, cannot decide now – revisit question later):

***The patient’s request for proxy if she/he cannot speak for her/himself:*** (ie. Unclear, cannot decide now – revisit question later):

***The patient’s wishes regarding information given to next of kin*** (ie. Knowing as much as possible, limited information, does not want to know, unclear, cannot decide now – revisit question later):

***The patient’s wish for future hospitalization*** (ie. Yes, no, unclear, cannot decide now – revisit later):

*Comments:*

***Other*** (ie. next of kin’s comments, living will etc.):

Date:

Signature:

***The patient’s wishes regarding participation in decisions about future treatment*** (ie. wants to make decisions themselves, discuss and make decisions along with family, physician informs thoroughly and helps with decision, physician makes decision, unclear, cannot decide now – revisit question later):

***The patient’s wishes regarding future treatment intensity*** (ie. Less or more extensive treatment, palliative treatment, unclear, cannot decide now – revisit question later):

***Any other needs or requests the patient has for the future?***
