# Supplementary material for: Implementing advance care planning in nursing homes – study protocol of a cluster-randomized clinical trial
Source: BMC Geriatr. 2018 Aug 13;18:180. doi: 10.1186/s12877-018-0869-1 (PMC6090595; doi:10.1186/s12877-018-0869-1)
Supplement: Supplementary file 7 — Data collection form. (DOCX 24 kb) [file 12877_2018_869_MOESM7_ESM.docx]

# Table 3. Registration form

Form number

Nursing home / Date of admittance

Date of registration

Age / Gender: Male / Female / Diagnoses (fill inn) / Religion: Christian / Muslim / Other / None / Unknown

During the past 12 months:

1. Has a conversation with the patient regarding end-of-life treatment been documented in the patient’s chart?

If yes: Was the patient’s competence to consent in this conversation assessed? If yes: was the patient found to be competent?

Is the following documented in the patient’s chart?

1. Anything the patient wants or wishes to experience in the future?
2. Anything the patient is worried about for the future?
3. Who is the patient’s chosen proxy?
4. What information does the patient want regarding him/herself?
5. What does the patient want next of kin /proxy to know about?
6. The patient’s wishes about participating in decision making regarding future treatment
7. Wishes regarding future treatment intensity (life-prolonging treatment^1^)

If yes for question 8, what is documented:

- Wishes expressed by patient
- Wishes of next of kin
- Next of kin’s knowledge of patient’s wishes
- Was the wish positive or negative to life-prolonging treatment?
- Other….

1. Wishes for future hospitalization

If yes for question 9, what is documented?

- Wishes expressed by patient
- Wishes of next of kin
- Next of kin’s knowledge of patient’s wishes
- Was the wish positive or negative to hospitalization?
- Other….

Is documentation regarding questions 1-9 readily available?

|  |  |
| --- | --- |

^1^ Here life-prolonging treatment refers to all treatment and measures taken to postpone the patient’s death. Examples of this could be resuscitation, other breathing help or heart-stimulating medication, fluids and nutrition treatment (intravenous or through tubes), dialysis, antibiotics or chemotherapy.

Written advance care planning/ Advance directive, filled out by patient him/herself

1. Does the patient have a living will that deals with treatment choices?

Decisions about treatment

1. Has the patient been given life-prolonging treatment in the nursing home in the last 12 months? / If yes, was competence to consent assessed? / Was the patient competent? / Was the treatment in line with the patient’s wishes? / Number of treatments: / Other:
2. Has the nursing home had the patient hospitalized in the last 12 months? / If yes, was competence assessed? / Was the patient competent? / Was the hospitalization in line with the patient’s wishes? / Number of hospitalizations: / Other:
3. Has life-prolonging treatment been withheld from the patient in the nursing home over the last 12 months? / If yes, was competence assessed? / Was the patient competent? / Was the decision in line with the patient’s wishes? / Number: / Other:
4. Has the nursing home decided not to hospitalize the patient in the last 12 months? If yes, was competence assessed? / Was the patient competent? / Was the decision in line with the patient’s wishes? / Number: / Other:

Comments:
