# Supplementary material for: Implementing advance care planning in nursing homes – study protocol of a cluster-randomized clinical trial
Source: BMC Geriatr. 2018 Aug 13;18:180. doi: 10.1186/s12877-018-0869-1 (PMC6090595; doi:10.1186/s12877-018-0869-1)
Supplement: Supplementary file 8 — Data collection form – in Norwegian. (DOCX 23 kb) [file 12877_2018_869_MOESM8_ESM.docx]

«Forberedende samtaler og felles planlegging av fremtidig helsehjelp»

**Registreringsskjema**

**Skjema nummer:**

| Sykehjem: | Dato for registrering: |
| --- | --- |
|  |  |
| Dato for innkomst sykehjem:  Innlagt fra: |  |

| Pasient | | | | |
| --- | --- | --- | --- | --- |
| Alder: | Kjønn:  Mann  Kvinne | Diagnose(r) (skriv inn): | Religion:  Kristendom  Islam  Annen  Ingen  Ukjent |  |
|  |  |  |  |  |

| Dokumentasjon av pasientens ønsker, fremtidig behandlingsintensitet og sykehusinnleggelse |
| --- |

| 1. Er det dokumentert samtale med pasienten om behandling ved livets slutt i pasientens journal? JA NEI   Hvis ja:  Ble pasientens samtykkekompetanse vurdert i forhold til samtalen(e)? JA NEI  Hvis ja – var pas. samtykkekompetent? JA NEI  Er følgende dokumentert i pasientens journal?   1. Noe pasienten håper på eller ønsker å oppleve for fremtiden. JA NEI 2. Noe pasienten uroer seg over for fremtiden. JA NEI 3. Hvem pasienten har oppgitt som nærmeste pårørende/stedfortreder. JA NEI 4. Pasientens ønsker om informasjon til seg selv. JA NEI 5. Pasientens ønsker om hva pårørende skal informeres om. JA NEI 6. Pasientens ønsker om å være med å bestemme ved fremtidig behandling. JA NEI 7. Ønsker om fremtidig behandlingsintensitet (livsforlengende behandling^1^). JA NEI   Hvis ja på spørsmål 8, hva er dokumentert:  - pasientens egne uttalte ønsker JA NEI  - pårørendes ønsker JA NEI  - pårørendes kunnskap om pasienters ønsker JA NEI  - annet: ……………………   1. Ønsker om fremtidig sykehusinnleggelse. JA NEI   Hvis ja på spørsmål 9, hva er dokumentert:  - pasientens egne uttalte ønsker JA NEI  - pårørendes ønsker JA NEI  - pårørendes kunnskap om pasienters ønsker JA NEI  - annet: ……………………  Er dokumentasjonen for spørsmål 1 -9 lett tilgjengelig? |
| --- |

^1^ Med livsforlengende behandling menes i denne sammenheng all behandling og alle tiltak som kan utsette en pasients død. Eksempel på dette er hjerte-lungeredning, annen pustehjelp og hjertestimulerende legemidler, ernærings- og væskebehandling (intravenøst eller gjennom svelg- eller magesonde PEG), dialyse, antibiotika og kjemoterapi.

| Skriftlig forhåndsønske utfylt av pasienten selv |
| --- |

| 1. Har pasienten skrevet et «livstestamente» (f.eks. fra foreningen retten til en verdig død) om behandlingsønsker? JA NEI VET IKKE |
| --- |

| Beslutning om behandling |
| --- |

1. Er livsforlengende behandling gitt til pasienten på sykehjemmet siste 12 måneder? JA NEI

Hvis ja, ble samtykke-kompetanse vurdert? JA NEI

Var pasienten samtykkekompetent? JA NEI

Var behandlingen i tråd med pasientens ønsker? JA NEI VET IKKE

Antall behandlinger:

Annet:

1. Har pasienten blitt innlagt fra sykehjemmet til sykehus siste 12 måneder? JA NEI

Hvis ja, ble samtykke-kompetanse vurdert? JA NEI

Var pasienten samtykkekompetent? JA NEI

Var sykehusinnleggelsen i tråd med pasientens ønsker? JA NEI VET IKKE

Antall innleggelser:

Annet:

1. Har livsforlengende behandling blitt besluttet ikke gitt til pasienten på sykehjemmet siste 12 måneder? JA NEI

Hvis ja, ble samtykke-kompetanse vurdert? JA NEI

Var pasienten samtykkekompetent? JA NEI

Var beslutningen i tråd med pasientens ønsker? JA NEI VET IKKE

Antall:

Annet:

1. Har sykehusinnleggelse fra sykehjemmet blitt besluttet ikke gitt siste 12 måneder? JA NEI

Hvis ja, ble samtykke-kompetanse vurdert? JA NEI

Var pasienten samtykkekompetent? JA NEI

Var beslutningen i tråd med pasientens ønsker? JA NEI VET IKKE

Antall:

Annet:

Kommentarer:
