# Supplementary material for: Implementing advance care planning in nursing homes – study protocol of a cluster-randomized clinical trial
Source: BMC Geriatr. 2018 Aug 13;18:180. doi: 10.1186/s12877-018-0869-1 (PMC6090595; doi:10.1186/s12877-018-0869-1)
Supplement: Supplementary file 9 — Changes to the project. (DOCX 13 kb) [file 12877_2018_869_MOESM9_ESM.docx]

# Changes to project during the intervention period

Here we describe more elaborately changes we made during the intervention period.

## Changes to implementation support

### Pocket card

The pocket card emphasized the importance of adapting the questions to the individual, and to seek a joint understanding of the discussion.

### Documentation template

The documentation template included space to make notes and the following headings: Those attending, assessment of patient DMC, something the patient wants to experience, patient’s worries for the future, general wishes for the future, patient’s wishes for: information to oneself, information to NOK, proxy, participation in future decision-making, and possible future life-prolonging treatment and hospitalization. We provided examples on how to document for each of the above-mentioned points. Any ambiguities in the patient’s responses were encouraged to be included in the documentation. For example, if life-prolonging treatment had been discussed with the patient and the patient did not have a clear idea on the subject, one of the pre-formulated recommendations on how to document was: “have not made up mind yet – should be followed-up later”.

## Changes to methodical issues

### Case-based interviews

In addition to the planned interviews, we interviewed one NOK and one physician individually to follow-up a case at one of the NHs. The researchers got information at a meeting with local project teams of ACP that affected treatment of a patient who died after hospitalization. The researchers decided ad-hoc to follow-up this case as it could illustrate how ACP in a NH may influence health care decisions at the hospital level.

### Chart review of patients who died

The background for including also those who died was primarily to analyze and compare the association of hospitalization and life-prolonging treatment towards end of life in the intervention and control group. We included patients who died during the last 9 months leading up to T0 and T1.

### Data collection from EHRs

We intended chart reviews to be the responsibility of the ward’s coordinator. However, TJLS performed all the registrations, except for the first NH visited where TJLS and EG did most of the registrations together. Project coordinators at the wards also gathered some information.
